# Gut Microbiota-Derived Small Extracellular Vesicles Endorse Memory-like Inflammatory Responses in Murine Neutrophils

**DOI:** 10.3390/biomedicines10020442

**Published:** 2022-02-14

**Authors:** Trim Lajqi, Natascha Köstlin-Gille, Stefan Hillmer, Maylis Braun, Simon A. Kranig, Stefanie Dietz, Christian Krause, Jessica Rühle, David Frommhold, Johannes Pöschl, Christian Gille, Hannes Hudalla

**Affiliations:** 1 Department of Neonatology, Heidelberg University Children’s Hospital, D-69120 Heidelberg, Germany; trim.lajqi@med.uni-heidelberg.de (T.L.); natascha.koestlin@med.uni-tuebingen.de (N.K.-G.); maylis.braun@med.uni-heidelberg.de (M.B.); simon.kranig@med.uni-heidelberg.de (S.A.K.); stefanie.dietz@med.uni-tuebingen.de (S.D.); christian.krause1995@gmx.de (C.K.); johannes.poeschl@med.uni-heidelberg.de (J.P.); christian.gille@med.uni-heidelberg.de (C.G.); 2 Department of Neonatology, University of Tübingen, D-72076 Tübingen, Germany; jessica.ruehle@med.uni-tuebingen.de; 3 Electron Microscopy Core Facility (EMCF), University of Heidelberg, D-69120 Heidelberg, Germany; stefan.hillmer@urz.uni-heidelberg.de; 4 Klinik für Kinderheilkunde und Jugendmedizin, D-87700 Memmingen, Germany; david.frommhold@klinikum-memmingen.de

**Keywords:** microbiota, neutrophils, small EVs, priming, inflammation, memory-like, transmigration, phagocytosis

## Abstract

Neutrophils are classically characterized as merely reactive innate effector cells. However, the microbiome is known to shape the education and maturation process of neutrophils, improving their function and immune-plasticity. Recent reports demonstrate that murine neutrophils possess the ability to exert adaptive responses after exposure to bacterial components such as LPS (Gram-negative bacteria) or LTA (Gram-positive bacteria). We now ask whether small extracellular vesicles (EVs) from the gut may directly mediate adaptive responses in neutrophils in vitro. Murine bone marrow-derived neutrophils were primed in vitro by small EVs of high purity collected from colon stool samples, followed by a second hit with LPS. We found that low-dose priming with gut microbiota-derived small EVs enhanced pro-inflammatory sensitivity as indicated by elevated levels of TNF-α, IL-6, ROS and MCP-1 and increased migratory and phagocytic activity. In contrast, high-dose priming resulted in a tolerant phenotype, marked by increased IL-10 and decreased transmigration and phagocytosis. Alterations in TLR2/MyD88 as well as TLR4/MyD88 signaling were correlated with the induction of adaptive cues in neutrophils in vitro. Taken together, our study shows that small EVs from stools can drive adaptive responses in neutrophils in vitro and may represent a missing link in the gut–immune axis.

## 1. Introduction

Neutrophils are terminally differentiated, short-lived effector cells and an important part of the innate immune system. They typically act as first responders to be recruited to the site of infection to eliminate pathogens by their armamentarium of antimicrobial agents (hydrolytic enzymes, proteinases, reactive oxygen species (ROS) and reactive nitrogen species (RNS), chemotactic agents and cytokines) [1,2,3]. Neutrophils have been incorrectly labeled as archaic immune cells with limited plasticity or immune-memory [4,5,6]. Accumulating reports demonstrate that this type of polymorphonuclear leukocyte contributes conjointly to a wide range of complex inflammatory disorders, highly dependent on disease circumstances and microbiome fluctuations [1,7].

Innate immune cells recognize different conserved pathogen-associated molecular patterns (PAMPs) as well as host-derived damage-associated molecular patterns (DAMPs) by various recognition receptors including Toll-like receptors (TLRs) [8,9]. As the first line of defense, they primarily promote rapid reactions against invading intruders through the production of several inflammatory factors (i.e., cytokines, chemokines) [10,11]. However, corresponding to other innate immune cell subsets such as monocytes, macrophages, natural killer (NK) cells and microglia, additional adaptive (memory-like) responses have been found in neutrophils. The capacity to boost their response to subsequent infection has been termed trained immunity [12,13,14,15]. Contrarily, the induction of tolerance is characterized by diminished responsiveness against secondary stimuli [16,17,18]. Initial studies showed that trained immunity and tolerance are PAMP-dependent; however, whereas priming with β-glucan (*C. albicans*), Bacille Calmette-Guérin (BCG), or oxidized low-density lipoprotein (oxLDL) promotes the release of pro-inflammatory mediators (i.e., IL-1β, IL-6, TNF-α, ROS), lipopolysaccharide (LPS) promotes an immune-suppressive phenotype with increased IL-10 production after a second stimulus [19,20,21,22,23,24,25]. Besides the activating agent, the magnitude of priming and the pathogen dose have subsequently been described as additional crucial factors for the regulation of adaptive responses [22,26,27,28,29,30,31,32]. Both training as well as tolerance are orchestrated by epigenetic modifications with resulting changes in metabolism [22,23,33,34,35,36,37].

Neutrophils have a short life span in the peripheral circulation, which makes in vitro studies challenging. However, the affinity of neutrophils to shape and regulate enduring immune responses has been reported [38,39,40,41,42]. Recently, we revealed that pathogenic stressors such as LPS (Gram-negative) and LTA (Gram-positive) promote the induction of opposing memory reactions in murine neutrophils [23,43,44]. Furthermore, a recent in vivo study showed that trained neutrophils express ROS-dependent anti-tumoral effects [45]. Microbiota may shape innate immune cell maturation [46,47,48] and have been reported to specifically navigate the education and maturation process of neutrophils [48,49]. In line with this, it was previously shown that microbiota depletion drives impaired granulopoiesis, resulting in functional and quantitative changes in neutrophils, provoking weakened resistance mechanisms [48,50]. Even though cross-talk between microbiota and neutrophils has been shown, there is to date no systematic analysis into the nature and function of this axis. Furthermore, the messenger of this cross-talk remains elusive. Cell-derived membranous structures known as extracellular vesicles (EVs), especially small EVs (sEV) with a diameter of 30–150 nm, are released from all living cells and can pass through various tissues. These nano-sized bio-packages contain a variety of nucleic acids, proteins and metabolites capable of altering biological responses in target cells [51,52,53]. They are also known to be engaged in the regulation of innate immune cells [23,51,54]. To our knowledge, however, there are no reports linking small EVs to the regulation of adaptive inflammatory features in innate immune cells.

We hypothesize that gut microbiota-derived small EVs could represent a missing link between the immuno-modulatory properties of the gut and peripheral immune cells such as neutrophils. As a first proof-of-principle in vitro study, we attempt to show the potential of gut-derived small EVs to prime bone marrow-derived neutrophils (BMDN) in a similar fashion, as was previously described for singular bacterial components such as LPS or LTA. Our data show that priming by low concentrations of small EVs after subsequent challenge with LPS triggers increased production of pro-inflammatory mediators (e.g., TNF-α, IL-6, ROS and MCP-1) and boosts transmigratory as well as phagocytic properties mediated particularly by the TLR2/MyD88 pathway. Contrarily, tolerant neutrophils primed by high concentrations of small EVs display an immuno-suppressive phenotype, characterized by weakened migratory as well phagocytic activity, mediated by both TLR2/MyD88 and TLR4/MyD88 signaling patterns. This study adds supporting evidence to the concept microbiota-mediated immune-memory of innate immune cells such as neutrophils.

## 2. Materials and Methods

### 2.1. Animals and Isolation of Bone Marrow Neutrophils

Adult (3–6 months old) wild-type C57Bl/6J mice (*n* = 36) were used for the isolation of bone marrow neutrophils. During the accommodation and for breeding purposes, all mice had free access to food and water and were kept in a 12 h light/dark cycle (IBF animal facility, University of Heidelberg). All experiments were approved by the regional authority (Karlsruhe Government Office, Germany (reference number: Az T-02/20)), and performed following EU guidelines (2010/63/EU Policy on the use of animals for scientific and educational purposes).

Wild-type mice were euthanized according to the approved protocol and femur and tibia were released from the connective tissue using scissors and a scalpel. Immediately after isolation, bones were moved to sterile conditions (cell culture bench) and processed by rinsing with 70% ethanol followed by repetitive washing steps with ice-cold 1x phosphate-buffered saline (1x PBS). Epiphyses of the bones were cut and the bone marrow was isolated by flushing the bones with 2 mM EDTA-RPMI-1640 medium (Ctlg. No. R8758-500 ML, Sigma-Aldrich, St. Louis, MO, USA) supplemented with 10% heat-inactivated FCS (Ctlg. No. PB-FCS-EU-0500, PeloBiotech, Planegg, Germany), 1% Penicillin-G/Streptomycin sulphate (Ctlg. No. Z-13-M, C.C. Pro, Oberdorla, Germany) and 1% Amphotericin B (Ctlg. No. A2942, Sigma-Aldrich, St. Louis, MO, USA). Neutrophils were isolated from the bone marrow by density gradient centrifugation using Percoll (Ctlg. No. 17-0891-02, GE Healthcare Bio-Sciences, Uppsala, Sweden) as reported previously [43,44,55]. Polymorphonuclear neutrophils were collected from the interface 64%/81%, subsequently washed by sterile PBS and cultivated in culture medium (supplemented RPMI-1640). Typically, isolated neutrophils had >95% viability (exclusion test) and showed >98% purity (microscopy by Hemacolor staining), as previously reported [55,56].

### 2.2. Isolation of Gut Microbiota-Derived Small EVs

**Figure 1:**
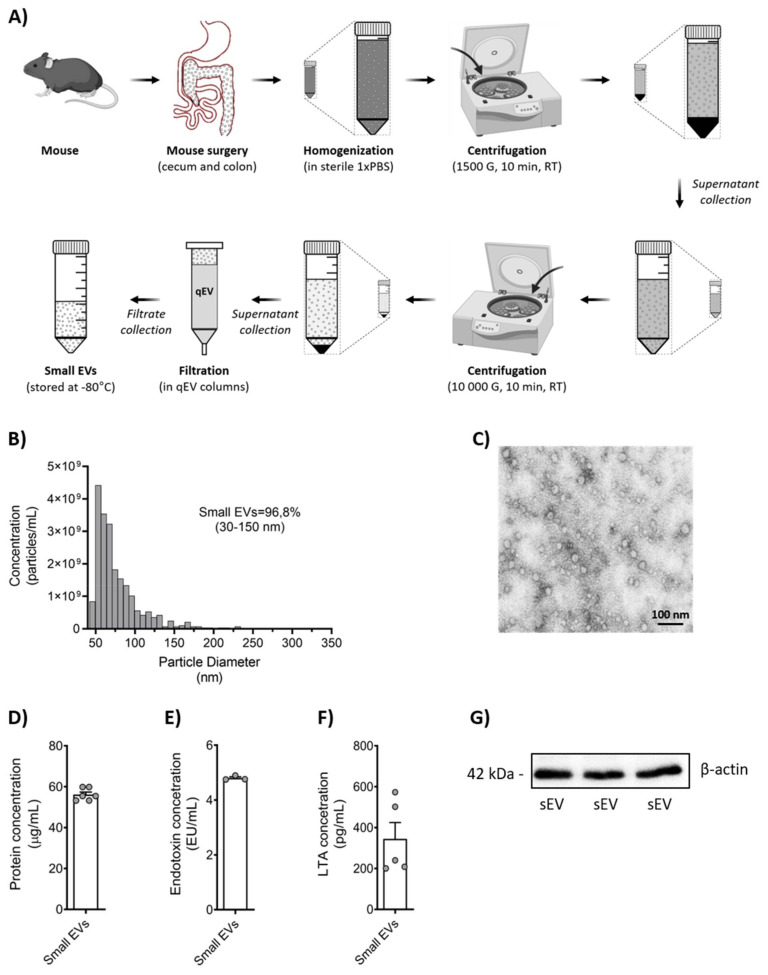
Schematic illustration of the isolation and purification process of small EVs from murine stool and their characterization. (**A**) Small EVs (sEV) isolated from the stool of mice from cecum and colon using qEV columns. Analysis of sEV concentration and their size distribution were determined by TRPS and TEM (**B**,**C**). Total protein concentration (**D**) was determined using the Pierce™ 660 nm Protein Assay Kit. Determination of endotoxin amount (**E**) was performed by Pierce LAL Chromogenic Endotoxin Quantification kit assay, whereas the amount of LTA (**F**) was measured by Mouse lipoteichoic acids (LTA) ELISA Kit. Protein expression of β-actin (**G**) was analyzed by Western blotting. Data are shown as scatter dot plots, mean + SEM (repeated measurements).

Stools were collected from the cecum and colon of *n* = 4 adult (3–6 months old) wild-type C57Bl/6J mice per experiment and pooled. Small EVs were isolated following recommendations from Izon Science LTD (Lyon, France), as shown in Figure 1A. Briefly, the stool samples were diluted in sterile Dulbecco’s Phosphate Buffered Saline (DPBS; Ctlg. No. D8537-500ML, Sigma-Aldrich, St. Louis, MO, USA) and homogenized. Then, samples were centrifuged at 1500× *g* for 10 min at room temperature (RT); the supernatant was collected and re-centrifuged at 10,000× *g* for 10 min at RT. Prior to filtration by qEV columns (Ctlg. No. SP5, qEV original −35 nm; Ser.:1001027, Izon Science LTD, Lyon, France), samples were sterile-filtered by a 0.2 µm filter (Ctlg. No. 83.1826.001, non-pyrogenic sterile Filtropur S 0.2. Sarstedt, Nümbrecht, Germany). Disruption of the qEV column flow was avoided since it may affect the accuracy of small EV separation. Small EVs were immediately stored at −80 °C. The total protein concentration was measured and used as the unit for further stimulation steps in this study.

### 2.3. Neutrophil Stimulation Protocol

**Figure 2:**
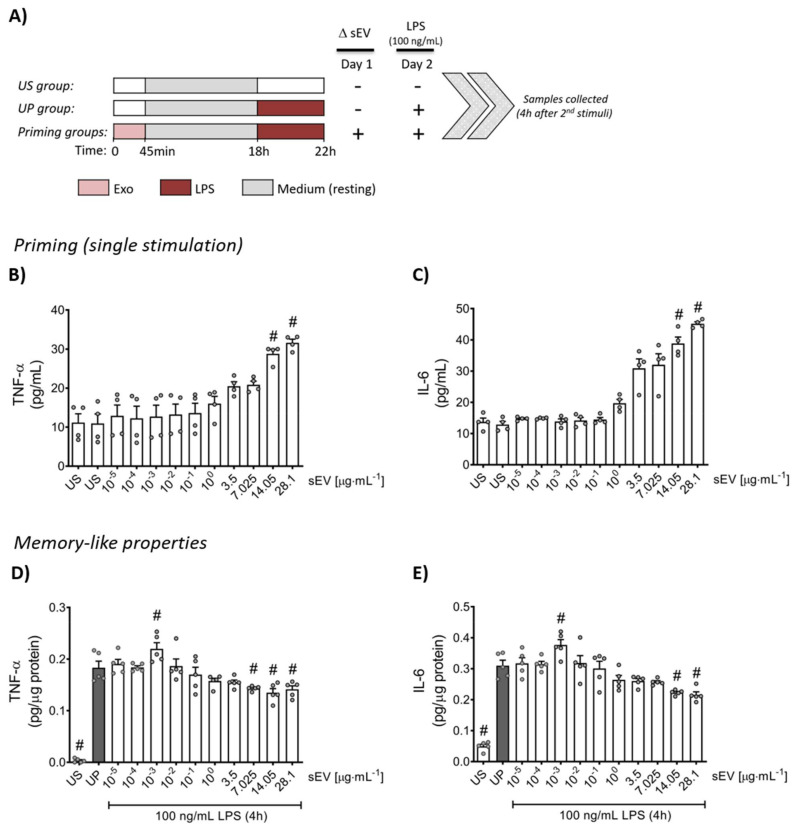
Cytokine responses with increasing concentrations of small EVs. (**A**) Bone marrow neutrophils were primed in a protein concentration-dependent manner with microbiota-derived small EVs ((**B**,**C**); *n* = 4) for 45 min and later after resting using the two-step protocol, re-challenged by 100 ng/mL LPS for 4 h expressing adaptive manners ((**D**,**E**); *n* = 5; normalized to total protein concentration). The cytokine production of TNF-α and IL-6 was measured using ELISA. Data are presented as scatter dot plots, mean + SEM, # *p* < 0.05, # versus unstimulated state (US) (**B**,**C**); # *p* < 0.05, # versus unprimed state (UP, gray bar) (**D**,**E**).

Shortly after isolation, neutrophils (10^6^ cells/well) were treated according to the two-step stimulation approach depicted in Figure 2A and as shown previously [43,44]. Cells were initially primed with increasing protein concentrations of isolated small EVs (first challenge: 10 pg/mL−28,1 µg/mL for 45 min) on day 1 and incubated at 37 °C and 5% CO_2_. The medium was subsequently changed, and cells were left to rest overnight (37 °C; 5% CO_2_). On day 2, neutrophils were re-challenged with a fixed dose (100 ng/mL) of LPS (E. coli 055:B5; Ctlg. No. tlrl-pb5lps, InvivoGen, Toulouse, France) for 4 h (37 °C; 5% CO_2_). Samples were collected 4 h after the second stimulus and processed for further analysis. Unstimulated (US) cells cultivated only in medium and unprimed (UP) cells (stimulated once on day 2 for 4 h with 100 ng/mL LPS) served as controls.

### 2.4. Characterization of Small EVs by Tunable Resistive Pulse Sensing (TRPS)

Isolated small EVs were characterized by their size, concentration and stability (zeta potential) using a tunable resistive pulse sensing (TRPS) technique from qNano (Izon Science, Christchurch, New Zealand). The analysis was performed using NP100 nanopores, capable of detecting particles within the size range of 50–300 nm (as stated by the manufacturer, Izon Science), and compared to the calibration beads CPC100 (mode diameter, 100 nm). Prior to the analyses, sEV samples were diluted 1:1 in 2x PBS supplemented with 0.1% Tween 20, as recommended by the manufacturer. Measurements were made with 44.29 mm of appropriate stretch and 0.44-volt (V) potential applied on the pores, with at least 500 particles being detected. Each measurement was run with a baseline current of 100 nanoamperes (nA) ± 10 nA. All samples were vortexed for 30 s and sonicated for 2 min prior to analysis. 

The charges were quantified by a four-calibration point using CPC100 as described [57,58,59].

### 2.5. Characterization of Small EVs by Transmission Electron Microscopy (TEM)

To visualize small EVs, the fractions were negatively stained. For negative staining, a glow-discharged carbon-coated (2 nm carbon) formvar grid was placed on a 20 μL drop of sample and allowed to adsorb to the carbon for 10 s. The sample was then washed three times briefly on a drop of water, stained with 3% *w/v* uranyl acetate in water, and dried. Micrographs were recorded using a transmission electron microscope (JEM 1400; JEOL Ltd, Freising, Germany) with a bottom-mounted high-sensitivity 4K CMOS camera (TemCam F416; TVIPS, Gauting, Germany) [60].

### 2.6. Measurement of Endotoxin Levels and Lipoteichoic Acid (LTA) Concentration

The concentration of Gram-negative endotoxin and Gram-positive lipoteichoic acid (LTA) in small EVs was measured using commercial enzyme-linked immunosorbent assay (ELISA) kits. Endotoxin levels were determined using Pierce LAL Chromogenic Endotoxin Quantitation Kit (Ctlg. No. 88282, Thermo Fisher Scientific, Waltham, MA, USA). Absorbance was measured at 405 nm in an iMark Microplate Reader (Bio-Rad Laboratories, Hercules, CA, USA). The endotoxin concentration was then calculated based on the values of the standard curve and expressed as endotoxin units per milliliter (EU/mL).

LTA levels were measured using Mouse lipoteichoic acids (LTA) ELISA Kit (Ctlg. No. MBS261639, MyBioSource, San Diego, CA, USA) according to the manufacturer’s instructions. Absorbance was read at 450 nm in an iMark Microplate Reader (Bio-Rad Laboratories, Hercules, CA, USA). The LTA concentration was then calculated based on the values of the standard curve and expressed as pg/mL.

### 2.7. Antibodies

Antibodies used in this study include: MyD88 (Ctlg. No. 4283), TLR2 (Ctlg. No. 13744) and TLR4 (Ctlg. No. 14358) purchased from Cell Signaling (Danvers, MA, USA). Antibody against β-actin (Ctlg. No. A5441, Sigma-Aldrich) served as loading control for Western blotting analysis. HRP-coupled anti-rabbit (Ctlg. No. 111-035-144, Dianova, Hamburg, Germany) and anti-mouse (Ctlg. No. 115-035-166, Dianova) were used as secondary antibodies.

### 2.8. SDS-PAGE Western Blotting

After the second challenge with LPS, bone marrow neutrophils were lysed using ice-cold 1x RIPA buffer containing freshly prepared phosphatase (10 mM Sodium orthovanadate) and protease (100 mg/mL Pefabloc; 1 mg/mL Pepstatin A; 1 mg/mL Leupeptin) inhibitors. Samples were vortexed and left for 1–2 min on ice, followed by centrifugation (30 min, 12,000 G at 4 °C). Supernatants were collected and stored at −20 °C or −80 °C. Prior to analysis, samples were mixed with 5× protein sample loading buffer (Laemmli buffer) containing sodium dodecyl sulfate (SDS) and then heated for 5–7 min at 95 °C. Samples were run on a 10% polyacrylamide gel and transferred to a polyvinylidenfluoride (PVDF) membrane. The membrane was blocked using 1% bovine serum albumin (BSA) for 30–45 min and primary antibodies were incubated overnight at 4 °C. After labeling with HRP secondary antibodies, a Chemi-Doc XRS+ camera (Bio-Rad Laboratories, Hercules, CA, USA) was used to capture protein bands. Data were quantified using Image Lab Ver. 6.0.1 software (Bio-Rad Laboratories, Hercules, CA, USA).

### 2.9. Assessment of the Total Protein Concentration

Cell lysates were analyzed for their protein content using Pierce™ 660 nm Protein Assay Reagent (Ctlg. No. 22662) supplemented with the ionic detergent compatibility reagent (IDCR; Ctlg. No. 22663) for broader detergent compatibility, both purchased from Thermo Fisher Scientific (Waltham, MA, USA). Then, 10 µL of the pre-diluted protein assay standards (Ctlg. No. 23208, Thermo Fischer Scientific, Waltham, MA, USA), samples and blanks were plated in a 96-well plate followed by immediate addition of the supplemented protein assay reagent. Absorbance was measured at 660 nm using an iMark Microplate Reader (Bio-Rad Laboratories, Hercules, CA, USA).

### 2.10. Cytokine and Chemokine Measurements

Supernatants were collected 4 h after LPS challenge and assessed for their cytokine (TNF-α, Ctlg. No. 430901; IL-6, Ctlg. No. 431301; and IL-10, Ctlg. No. 431411) and chemokine (MCP-1, Ctlg. No. 432701) levels using commercial ELISA kits purchased from BioLegend (San Diego, CA, USA) [22,26,43]. Using an iMark plate reader (Bio-Rad Laboratories, Hercules, CA, USA), absorbance was measured at 450 nm, subtracting absorbance at 570 nm (reference wavelength). The cytokine and chemokine production levels after the second stimulation were normalized to the total protein concentrations and presented as pg/µg of total protein.

### 2.11. Measurement of Reactive Oxygen Species (ROS)

Murine neutrophils (10^5^ cells/well) were stimulated in a dark, clear bottom 96-well plate according to the protocol described before (Figure 2A) [43,44]. Four hours after the LPS challenge, neutrophils were resuspended in 20 µM 2’,7’-dichlorofluorescin diacetate (DCFDA; Ctlg. No. ab113851, Abcam, Cambridge, UK) solution and incubated under dark conditions (30 min at 37 °C). The fluorogenic DCFDA that enters the living cells is initially deacetylated by cellular esterases to a non-fluorescent compound and then oxidized by ROS into a highly fluorescent dye, 2′,7′-dichlorofluorescein (DCF). Fluorescence (Ex/Em = 485 nm/535 nm) analysis was performed in a PerkinElmer Wallac Victor3 plate reader (PerkinElmer Life and Analytical Sciences, Turku, Finland).

### 2.12. In Vitro Transmigration Assay

Transmigration was assessed by a 3 µm pore size Cell Biolabs migration assay kit (Ctlg. No. CBA-104, San Diego, CA, USA) [43,44]. Briefly, murine neutrophils (10^5^ cells/well) 4 h after LPS challenge were resuspended in FCS-free medium and placed in the upper chamber (membrane chamber). The suspension of cells was left to migrate through the polycarbonate membrane for 5 h (37 °C; 5% CO_2_) into the bottom side (feeder tray) containing 10% FCS-enriched medium. Next, neutrophils remaining in the bottom side of the membrane chamber were dissociated using a pre-warmed Cell Detachment buffer (30 min; 37 °C). Finally, migrated cells were lysed by 4x lysis buffer and quantified using CyQuant GR fluorescent dye (20 min; room temperature). Fluorescence measurements (Ex/Em = 485 nm/535 nm) were performed using a PerkinElmer Wallac Victor3 plate reader (PerkinElmer Life and Analytical Sciences, Turku, Finland) and data were presented as relative fluorescence units (RFU).

### 2.13. In Vitro Phagocytosis Assay

The phagocytic capacity of bone marrow neutrophils was analyzed using enzyme- labeled E. coli particles purchased from Cell Biolabs (Ctlg. No. CBA-222, San Diego, CA, USA) [43]. Next, 10^5^ cells/well after LPS challenge were incubated with 10 µL of E. coli suspension and incubated for 4 h (37 °C; 5% CO_2_) followed by several washing steps and fixated according to the manufacturer’s instructions. Next, the plate was incubated for 30 min with blocking buffer (100 µL/well) at room temperature, washed thrice with PBS and the cells were permeabilized (100 µL/well) for a short duration (5 min; room temperature) and washed again using PBS. Afterwards, the plate was incubated for 30 min with substrate solution (100 µL/well) to initiate the reaction and, finally, the reaction was stopped by pipetting 100 µL/well stop solution provided in the kit. The absorbance was read at 450 nm using an iMark plate reader (Bio-Rad Laboratories, Hercules, CA, USA), and the data are expressed as optical density (OD) values. 

### 2.14. Analysis of Cell Viability-MTT Assay

Murine neutrophils (2 × 10^5^ cells/well) were plated in a 96-well plate and assayed for their viability by the MTT method. After the LPS challenge, cells were incubated with MTT (Thiazolyl Blue tetrazolium bromide; final conc. 0.5 mg/mL) reagent solution and incubated for 4 h (37 °C; 5% CO_2_), followed by overnight incubation after adding the solubilization solution. Lastly, the absorbance values were read at 570 nm using an iMark plate reader (Bio-Rad Laboratories, Hercules, CA, USA). The unprimed state was assigned as 100% and data are expressed as relative viability of murine neutrophils (Supplementary Figure S2).

### 2.15. RNA Isolation and Real-Time qPCR

Neutrophils were lysed using TRIsure Lysis Reagent (Ctlg. No. BIO-38032, Bioline, Luckenwalde, Germany) as described [61]. Prior to the synthesis of complementary DNA (cDNA), the quality and total RNA concentration were analyzed (Nanodrop DS11 FX+, DeNovix, Wilmington, DE, USA). High-Capacity cDNA Reverse Transcription kits (Ctlg. No. 4368814, Applied Biosystems) were used for cDNA synthesis and samples were analyzed by real-time qPCR (StepOnePlus, Applied Biosystems, Waltham, MA, USA). Primer pairs and their sequences are listed in Table 1.

### 2.16. Statistical Analysis

Statistical analysis was performed using SigmaPlot Software ver. 12.0 (Systat Software GmbH, Erkrath, Germany) and the graphs were processed by GraphPad Prism Software ver. 8.0.2 (GraphPad Software, San Diego, CA, USA). Data are presented as scatter dot plots and mean + SEM. A Shapiro–Wilk normality test was performed prior to the usage of one-way analysis of variance (ANOVA) test. Comparison between experimental groups was performed using one-way ANOVA. If the normality test failed, a Kruskal–Wallis test on ranks was used. Post hoc comparisons were performed by the Holm–Sidak test or Dunn’s method, as appropriate. A *p* < 0.05 was considered significantly different.

## 3. Results

### 3.1. Murine Microbiota-Derived Small EV Purification and Characterization

Microbiota-derived small EVs were isolated from adult mice as illustrated in Figure 1A. TRPS analysis for size and concentration revealed high quality with about >96% small EVs (Figure 1B) and constant electrostatic stability (<−10 mV) (Supplementary Figure S1).

TEM showed that the isolated small EVs were spherical and appeared to have a closed membrane bilayer (Figure 1C). Analysis of protein concentration was determined and used as a standard unit for sEV dosing throughout the study (Figure 1D). Western blot analysis for β-actin was used as a second method to demonstrate protein content in our sEVs (Figure 1G). As to be expected, we found both LTA and endotoxin in our sEV preparation (Figure 1E,F).

### 3.2. Microbiota-Derived Small EVs Promote Dose-Dependent Memory-like Inflammatory Features in Murine Bone Marrow Neutrophils

Priming with increasing concentrations of microbiota-derived small EVs showed a concentration-dependent increase in the concentration of the cytokines, TNF-α and IL-6 (Figure 2B,C). Resting cells were re-challenged 18 h after the priming step with LPS (100 ng/mL) for 4 h. Low concentration (1 ng/mL) sEV priming led to elevated levels of IL-6 and TNF-α contrary to high concentration (28.1 µg/mL) priming featuring suppressed cytokine levels (Figure 2D,E).

**Figure 3:**
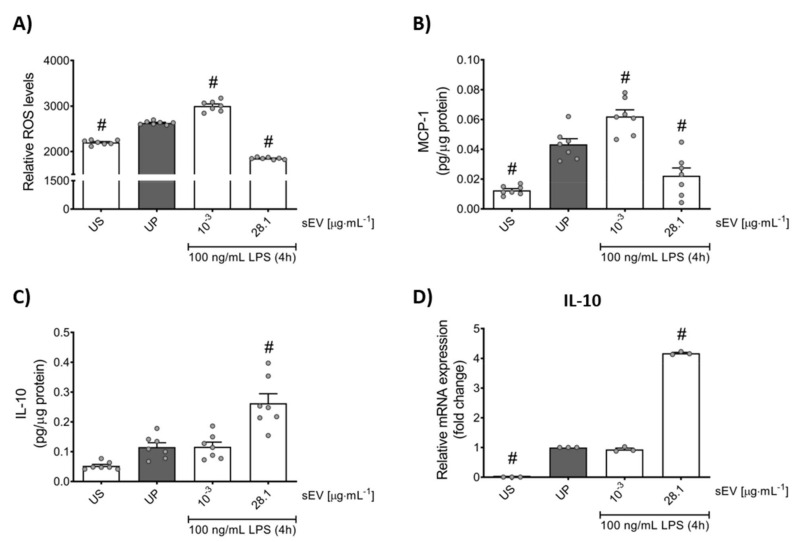
Role of microbiota-derived small EVs altering pro- and anti-inflammatory mediators in bone marrow neutrophils. Murine bone marrow neutrophils were primed for 45 min by microbiota-derived small EVs (low concentration: 1 ng/mL; high concentration: 28,1 µg/mL) and re-challenged with 100 ng/mL LPS for 4 h on day 2. Production of (**A**) ROS (*n* = 7) was determined by DCFDA assay, whereas (**B**) MCP-1 (*n* = 7) and (**C**) IL-10 (*n* = 7) were measured by ELISA (normalized to total protein concentration). Real-time qPCR was used to analyze the gene expression of (**D**) IL-10 (*n* = 3; unprimed state assigned as 1.0). Data are presented as scatter dot plots, mean + SEM, # *p* < 0.05, # versus unprimed condition (UP, gray bar).

To further elaborate the role of microbiota-derived small EVs promoting memory-like reactions in murine neutrophils, we broadened the panel of inflammatory markers analyzing the production of ROS and MCP-1 as a pro-inflammatory and IL-10 as an anti-inflammatory mediator (Figure 3). Trained neutrophils (1 ng/mL) exhibited elevated levels of ROS as well as MCP-1, whereas murine-tolerant neutrophils (28.1 µg/mL) displayed diminished responses. 

In contrast to pro-inflammatory mediators, the release of IL-10 was enhanced by high-protein (28.1 µg/mL) concentration sEV priming, but was not affected by low-concentration sEV priming (Figure 3C,D).

To exclude different cell survival between the treatment groups as a confounder, we analyzed the cell viability of murine neutrophils using the MTT assay, showing no differences in cell viability between groups (Supplementary Figure S2).

### 3.3. Small EV-Priming Is Promoted by TLR2/MyD88 Activation

**Figure 4:**
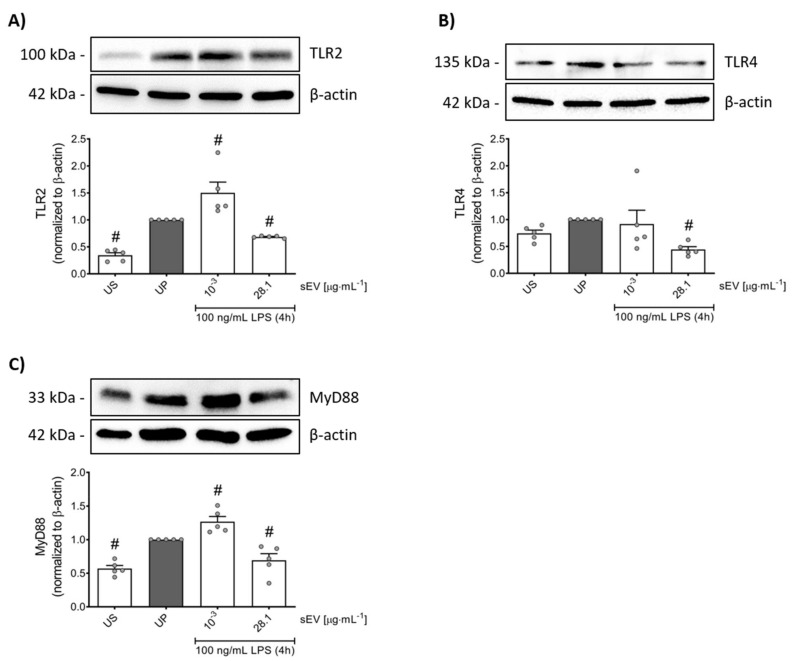
Signaling events behind gut microbiota-derived small EVs in murine neutrophils. Murine bone marrow neutrophils were primed for 45 min by microbiota-derived small EVs (low concentration: 1 ng/mL; high concentration: 28.1 µg/mL) and later, on day 2, were re-challenged by 100 ng/mL LPS for 4 h as described above. Protein expression of TLR2 (**A**, *n* = 5), TLR4 (**B**, *n* = 5) and MyD88 (**C**, *n* = 5) were assayed by Western blotting and quantified (unprimed cells assigned as 1.0). Data are presented as scatter dot plots, mean + SEM, # *p* < 0.05, # versus unprimed condition (UP, gray bar).

Since our sEV preparation was shown to originate from both Gram-positive and Gram-negative bacteria, we decided to verify the main signaling events involving the activation of Toll-like receptors (TLRs). Our data indicate that the LPS challenge of neutrophils after low-concentration sEV priming correlates with elevated expression levels of the TLR2/myeloid differentiation primary response protein 88 (MyD88) pathway without affecting TLR4 (Figure 4). 

Distinct from trained neutrophils, tolerant neutrophils primed by high concentrations of small EVs exhibited declined levels of the TLR4/TLR2/MyD88 trinity (Figure 4).

### 3.4. Small EV-Priming Alters Migratory Activities and Phagocytic Capacity of Murine Bone Marrow Neutrophils

**Figure 5:**
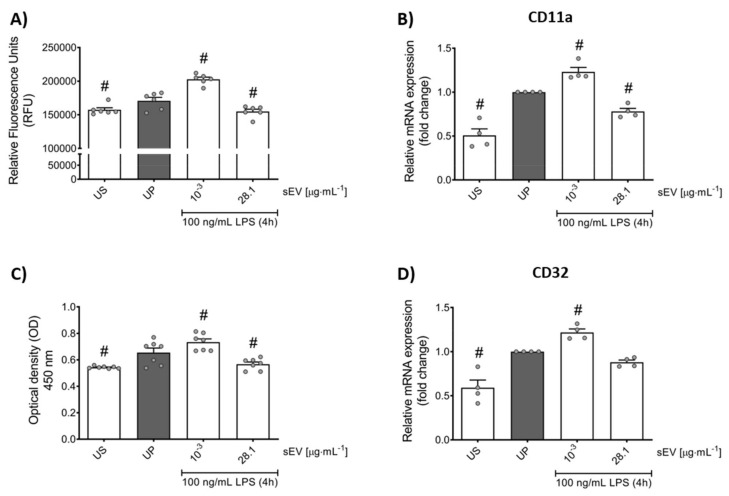
Microbiota-derived small EVs reshape migratory effects and phagocytic activity in murine neutrophils. Neutrophils were primed for 45 min by microbiota-derived small EVs (low concentration: 1 ng/mL; high concentration: 28.1 µg/mL) and re-challenged by 100 ng/mL LPS for 4 h on day 2. Migratory activities of neutrophils (**A**, *n* = 6) were analyzed using commercially available kits as described above and data were shown as relative fluorescence units (RFU), whereas real-time qPCR was used to analyze the gene expression of (**B**) CD11a (*n* = 4) and (**D**) CD32 (*n* = 4) (unprimed state assigned as 1.0). Phagocytic activity (**C**, *n* = 7) of murine neutrophils was determined by commercially available kits as described above and data were expressed as optical density (OD) values. Data are presented as scatter dot plots, mean + SEM, # *p* < 0.05, # versus unprimed condition (UP, gray bar).

Next, we validated whether priming by small EVs translates into biological function. Low-concentration sEV priming promotes increased transmigration in murine neutrophils in vitro, supported by respective expression of CD11a as an integral regulator of cell recruitment (Figure 5A,B). Further, trained neutrophils exhibited elevated phagocytic efficiency endorsed by its surface receptor CD32 that regulates both phagocytosis and cytokine release (Figure 5C,D). Tolerant neutrophils displayed the opposite functional effects.

## 4. Discussion

Our knowledge on innate immune-memory or trained immunity is still evolving. Innate memory most likely represents an evolutionary adaptation of innate immune cells to promote resistance mechanisms against a broad spectrum of infectious agents [63]. A growing number of reports have identified several factors influencing the induction of adaptive responses in innate immune cells, such as pathogen-specificity (β-glucan, BCG, oxLDL, LPS), pathogen dose (low vs. high) and magnitude of priming [19,20,21,22,23,27,43,64]. Epigenetic modifications involving H3 or H4 histones with resulting changes in metabolism have been reported to arrange the development of adaptive features in innate immune cells [20,22,33,34,35,36].

We also know that gut microbiota shape immune-responses; however, the specific signaling and means of communication remain elusive. Several studies have revealed that nano-sized membrane vesicles derived from Gram-negative as well Gram-positive bacteria may trigger potent inflammatory reactions [65,66,67,68,69]. Additionally, bacterial biovesicles may drive differential biological effects mediated by specific molecular cargo, depending on the formation route [70,71,72]. Small EVs represent such cell-secreted nanocarriers containing proteins, lipids, metabolites and nucleic acids (DNA, RNA) [51,73]. Due to their advanced abilities to carry several bioactive components, possessing additionally low immunogenicity and demonstrating excellent cell infiltration ability, this type of biovesicle holds promising therapeutic potential [73,74]. As downsides, they are also characterized by rapid clearance, precarious loading efficiency and poor stability ex vivo [73,75,76]. Small EVs are secreted by all cell types and can be collected from numerous compartments and biomaterials (i.e., blood, urine, stool (feces), cerebrospinal fluid). 

The current study identifies gut microbiota-derived small EVs as possible mediators regulating the development of adaptive properties in murine bone marrow neutrophils in vitro. The intestinal lumen largely consists of bacteria that live in a mutual symbiotic relationship with the host [77,78]. Naturally, the amount of different bacterial compounds, mediators and metabolites and their effect on the host organism are vast. We have previously shown that individual components such as LPS and LTA, which are also present in the current small EV preparation, may prime neutrophils in vitro. In the current work, we focus on biological function and plausibility rather than specific molecular mediators. Our data support the notion of a direct interplay between microbiota and innate immune cells, more specifically the induction of training or tolerance of neutrophils. Characterization studies performed on gut microbiota-derived small EVs show that they are likely derived from both Gram-negative and Gram-positive bacteria. Akin to LPS or LTA, neutrophil priming by gut-derived small EVs supports pro- or anti-inflammatory properties depending on the actual dose [43,44]. This binary regulation has been shown for other innate immune cells such as monocytes, macrophages and microglia [22,27,29,79]. 

The question remains whether these in vitro observations are also translatable to a living organism. Recently, Kalafati et al. showed that neutrophil-induced trained immunity in vivo supports tumor suppression in a ROS-dependent manner [45,80]. In line with these findings, several studies suggested that trained cells, especially neutrophils and monocytes, exhibit a pro-inflammatory phenotype with increased antimicrobial activity outlined by heightened transmigration and phagocytosis [25,81,82]. In general, bacterial EVs may mediate the regulatory properties of their host cells [83]. More specifically, gut-derived bacterial EVs have been shown to mediate many immune modulatory properties of the microbiome [84,85] and have been suggested as an alternative to probiotics in immune-compromised individuals [86]. Our study supports this novel mechanism of communication between the gut and immune cells. Potentially, this mechanism might help link alterations in microbiome composition to neutrophil-mediated pathology like for example cystic fibrosis (CF). It was shown that EVs from CF patients differ from healthy controls in terms of composition and quantity and EVs from epithelial CF cell lines may activate neutrophils [87]. Interestingly, the study in CF patients highlighted an increase in RAGE-mediated MAPK signaling in neutrophils, which was partially mediated by EV cargo (calcium-binding protein S100A12). A similar effect on neutrophils was shown for hypoxia-induced endothelial release of EVs containing S100A12 [88]. Children with CF show an altered microbiome composition [89,90], and even though the link has not been sufficiently explored, it is plausible that gut-derived EVs also affect neutrophil function.

Various studies have shown that Gram-negative as well Gram-positive bacteria follow distinct routes of vesicle formation, resulting in different architectural envelopes [70,72,91]. LPS, especially its hydrophobic anchor, lipid A, serves as a potent activator of TLR4 and as an exterior leaflet of Gram-negative bacterial vesicles, whereas Gram-positive bacterial vesicles are characterized by their LTA presence on the surface engaging the activation of TLR2 [71,72]. Investigation of signaling patterns revealed that training by low concentrations of small EVs is mediated by TLR2/MyD88 upregulation, suggesting an LTA regulation of innate memory by small EVs in murine neutrophils. Contrary to trained neutrophils, the induction of tolerance by high-protein-concentration small EVs is driven by downregulated protein expression of both signaling patterns: TLR2/MyD88 as well as TLR4/MyD88. Likewise, we previously demonstrated that low-dose LTA may endorse memory-like responses such as training of murine neutrophils, whereas high-dose primed neutrophils by LTA or LPS trigger the development of an anti-inflammatory phenotype [43,44]. The involvement of TLR2 as well as TLR4 in the mediation of inflammatory reaction in innate immune cells regulating cellular functions such as transmigration and phagocytosis has been proclaimed in numerous studies [92,93,94,95,96,97]. 

Several limitations to our study need to be acknowledged. First off, we are using an in vitro assay on bone marrow-derived neutrophils. Even though the purity of neutrophils is generally high, BMDN differ from peripheral neutrophils and the observed regulatory effect of gut small EVs might not be the same in this particular population. Additionally, the origin of small EVs in our preparation remains entirely unknown. As all cells secrete EVs, it is notoriously difficult to delineate the origin in mixed cellular compartments [98]. The sEV population in the current study might stem from both bacterial and mammalian cells. Even though the pattern of priming as well as the down-stream signaling after exposure to EVs is very similar to priming with LPS or LTA, it remains mere speculation which subgroup of EVs is responsible for the observed biological effect. To complicate the matter, it was also shown that mammalian cells such as M. tuberculosis-infected macrophages may produce both CD9/CD63 positive and bacterial EVs [99]. Definitive answers as to the source of bioactive EVs could only result from in vitro cultures of gut bacteria; however, the complexity of the gut microbiome can hardly be mimicked in a dish and removing the host as a regulatory entity raises novel limitations, which are almost impossible to address. In order to delineate the effect of bacterial and mammalian EVs, Park et al. used fecal EVs from germ-free and wild-type (wt) mice [68]. Only wt-EVs were capable to mount sepsis-like reactions and TLR2 and TLR4 activation. However, specific cell types or immune priming were not addressed. Furthermore, there is an ongoing debate about the specific cargo of small EVs, and even though we can show that our preparation contain LPS and LTA, their relevance and function in the current context are not clear [51,52,91,100,101]. Of note, the translational limitation of murine findings to human immunology should be highlighted. Neutrophils show species-specific function, morphology and physiology due to their different gene expression patterns, especially guiding the release of cytokines and chemokines [102,103,104]. 

## 5. Conclusions

Our study is the first to describe a direct interplay between gut microbiota-derived small EVs and neutrophils and their potential to modulate neutrophil function and immune-memory in vitro. The dose of small EVs appears to regulate priming towards opposing immunological states, where a low concentration of small EVs endorses training effects, whereas high concentrations support immune tolerance in murine neutrophils. This observation adds another missing piece to the puzzle of the gut–immune axis in neutrophils. The translational potential of gut microbiota-derived small EVs modulating neutrophil function and plasticity should be drawn with caution and warrants further investigation.

## Figures and Tables

**Table 1:** Primer pairs and their sequences.

Gene Name		Primer Sequence (5′–3′)
**IL-10** (Interleukin 10)	Forward: Reverse:	ACCAGCTGGACAACATACTGC TCACTCTTCACCTGCTCCACT
**CD11a** (Integrin α-L)	Forward: Reverse:	AGATCGAGTCCGGACCCACAG GGCAGTGATAGAGGCCTCCCG
**CD32** (Cluster of differentiation 32)	Forward: Reverse:	AATCCTGCCGTTCCTACTGATC GTGTCACCGTGTCTTCCTTGAG
**GAPDH** (Glyceraldehyde-3-phosphate dehydrogenase)	Forward: Reverse:	CATGGCCTTCCGTGTTTCCTA CCTGCTTCACCACCTTCTTGAT

GAPDH was used as house-keeping gene. Relative gene expression was calculated using the comparative C_T_ (2^−ΔΔC^_T_) method [62].

## Data Availability

The data and materials demonstrated in this study are available upon a reasonable request.

## Supplementary Materials

Supplementary materials can be accessed online at https://www.mdpi.com/article/10.3390/biomedicines10020442/s1, Supplementary Figure S1: Characterization of zeta potential and population of microbiota-derived small EVs by TRPS. Supplementary Figure S2: Analysis of cell viability of murine neutrophils primed by microbiota-derived small EVs.
