# Supplementary material for: Gut Microbiota-Derived Small Extracellular Vesicles Endorse Memory-like Inflammatory Responses in Murine Neutrophils
Source: Biomedicines. 2022 Feb 14;10(2):442. doi: 10.3390/biomedicines10020442 (PMC8962420; doi:10.3390/biomedicines10020442)
Supplement: Supplementary file 1 [file biomedicines-10-00442-s001.zip › biomedicines-1562102-supplementary.pdf]

biomedicines

MDPI

Supplementary Figures

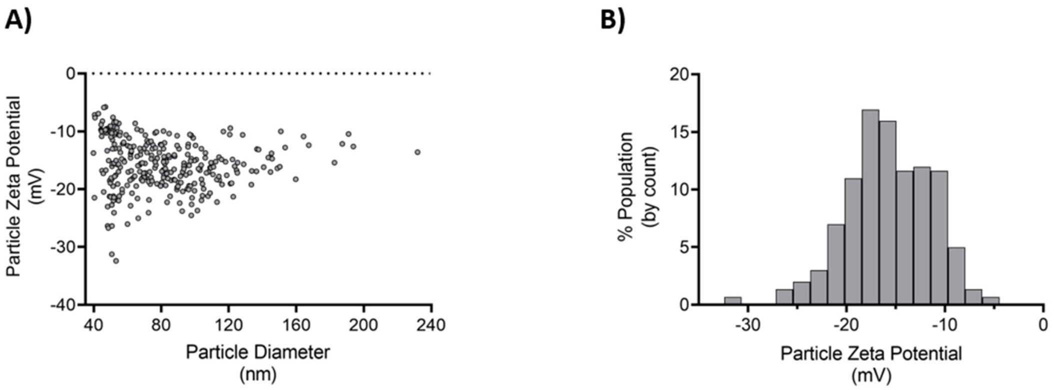

Supplementary Figure S1. Characterization of zeta potential and population of microbiota-derived small EVs by TRPS. Small EVs isolated from the stool of mice from the caecum and colon using qEV columns were further analyzed for their (**A**) zeta potential and (**B**) population distribution by TRPS technique. Data are shown as XY scatter plots.

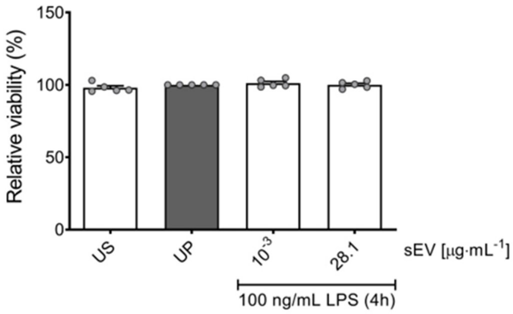

Supplementary Figure S2. Analysis of cell viability of murine neutrophils primed by microbiota-derived small EVs. Murine bone marrow neutrophils were primed for 45 min by microbiota-derived small EVs (low concentration: 1 ng/mL; high concentration: 28,1 µg/mL) and re-challenged by 100 ng/mL LPS for 4h on day 2. Analysis of (**A**) cell viability (n=5) was performed by MTT assay, as described in the method section. Data are shown as scatter dot plots (UP group assigned as 100%), mean + SEM.
